# Structure of the *Drosophila* nucleosome core particle highlights evolutionary constraints on the H2A-H2B histone dimer

**DOI:** 10.1002/prot.21720

**Published:** 2007-10-23

**Authors:** Cedric R Clapier, Srinivas Chakravarthy, Carlo Petosa, Carlos Fernández-Tornero, Karolin Luger, Christoph W Müller

**Affiliations:** 1 European Molecular Biology Laboratory, Grenoble Outstation38042 Grenoble Cedex 9, France; 2 Howard Hughes Medical Institute and Department of Biochemistry and Molecular Biology, Colorado State UniversityFort Collins, Colorado 80523-1870; 3 European Molecular Biology Laboratory, Structural and Computational Biology UnitMeyerhofstrasse 1, Heidelberg D-69117, Germany

**Keywords:** chromatin, nucleosome core particles, protein–DNA interaction, *Drosophila*, crystal structure

## Abstract

We determined the 2.45 Å crystal structure of the nucleosome core particle from *Drosophila melanogaster* and compared it to that of *Xenopus laevis* bound to the identical 147 base-pair DNA fragment derived from human α-satellite DNA. Differences between the two structures primarily reflect 16 amino acid substitutions between species, 15 of which are in histones H2A and H2B. Four of these involve histone tail residues, resulting in subtly altered protein–DNA interactions that exemplify the structural plasticity of these tails. Of the 12 substitutions occurring within the histone core regions, five involve small, solvent-exposed residues not involved in intraparticle interactions. The remaining seven involve buried hydrophobic residues, and appear to have coevolved so as to preserve the volume of side chains within the H2A hydrophobic core and H2A-H2B dimer interface. Thus, apart from variations in the histone tails, amino acid substitutions that differentiate *Drosophila* from *Xenopus* histones occur in mutually compensatory combinations. This highlights the tight evolutionary constraints exerted on histones since the vertebrate and invertebrate lineages diverged.

## INTRODUCTION

Genomic DNA in the eukaryotic nucleus is compacted and organized in protein–DNA complexes called chromatin. The notion of a repeating unit of chromatin structure, composed of eight histone proteins and ∼200 base pairs of DNA, was proposed over 30 years ago.^1^,^2^ Within this unit, the first level of chromatin organization, revealed by micrococcal nuclease digestion, is the nucleosome core particle (NCP). The NCP is composed of an octamer containing two copies of each of the four histone proteins (H3, H4, H2A, and H2B), around which ∼146 base pairs of DNA are tightly wrapped in 1.65 turns of a left-handed superhelix (reviewed in Ref.^3^).

In addition to its structural role in genome organization, the nucleosome is the point of convergence for many DNA regulatory processes: recombination, repair, replication, and transcription. In particular, nucleosomes are highly dynamic and are directly involved in the regulation of transcription.^4^ ATP-dependent remodeling complexes physically modulate chromatin structure at the nucleosome level, actively altering the accessibility of specific sequences to transcription factors.^5^,^6^ Nucleosomes also carry information via changes in composition (histone variants) and posttranslational modifications (PTMs).^7^,^8^ The role of both types of modification on the regulation of genomic activity is currently the subject of intense research. Finally, the nucleosome is involved in major cellular regulatory mechanisms related to cell cycle and aging, cell differentiation, and cellular reprogramming,^9^ and plays a critical role in viral infection^10^ and cancer.^11^

Structural studies of the nucleosome over the last 20 years have painted an increasingly accurate picture of how the nucleosome accomplishes its packaging and regulatory roles. Initial low-resolution studies^12^–^14^ followed by the crystal structure of the histone octamer^15^ elucidated the overall architecture of the NCP. The structure of an NCP (Xla-NCP146) composed of recombinant *Xenopus laevis* histones and a 146-bp palindromic fragment of human α-satellite DNA revealed details of the DNA structure and its interactions with the histones.^16^ A higher resolution structure (Xla-NCP147) using a related 147-bp DNA fragment allowed for a detailed analysis of the DNA conformation, solvent structure, and interactions with ions.^17^–^19^ Structures of a *Xenopus* NCP containing the histone variant H2A.Z^20^ or macroH2A^21^ and of NCPs comprising chicken,^22^ yeast,^23^ and human histones^24^ have brought additional functional and evolutionary insights.

To extend this analysis, we determined the crystal structure of the NCP from *Drosophila melanogaster*, the first from an invertebrate species. *Drosophila* histones share a high degree of sequence identity with those of *Xenopus*, ranging from 83% and 89% identity for H2B and H2A, respectively, to 99% for H3 and H4. Most of these changes localize to histone tail residues that are disordered in the available NCP crystal structures. However, a substantial number involve structured histone residues. We compare the *Drosophila* and *Xenopus* NCP structures and focus particularly on histone residues that have diverged between these species.

## MATERIALS AND METHODS

### Crystallization

NCPs were prepared from recombinant *D. melanogaster* histones and a 147 bp palindromic DNA fragment derived from human α-satellite DNA, as described previously.^25^ Crystallization trials were carried out by the hanging drop vapor-diffusion technique at 4°C by equilibrating a droplet containing 3 mg/mL Dm-NCP147, 80–85 m*M* MnCl_2_, 50–80 m*M* KCl, and 20 m*M* potassium cacodylate (pH 6.0) against a reservoir solution containing of 40–42.5 m*M* MnCl_2_, 25–40 m*M* KCl, and 20 m*M* potassium cacodylate (pH 6.0). To improve diffraction quality, crystals were soaked overnight in the reservoir solution supplemented with 24% (v/v) 2-methyl-2,4-pentanediol as cryoprotectant and flash-cooled in liquid nitrogen.

### Crystallography

Diffraction data were collected at ESRF beamline ID14-3 (λ = 0.931 Å) on a MAR CCD detector and processed with XDS^26^ and programs of the CCP4 suite.^27^ Crystals obtained using described conditions^17^ were isomorphous to the Xla-NCP147 crystal form (Table I). The Xla-NCP147 structure (pdb id 1KX5) minus the N-terminal histone tail residues was used as a starting model. Positioning this model into the Dm-NCP147 unit cell resulted in a crystallographic *R*-factor of 0.40, which dropped to 0.32 upon rigid body refinement using CNS.^28^ A further round of restrained coordinate and *B*-factor refinement reduced this to 0.276 (*R*_free_ = 0.301). Differences between the *Drosophila* and *Xenopus* structures were readily apparent in a 2*F*_o_ − *F*_c_ map calculated using phase information from the Xla-NCP147 atomic coordinates. Iterative rounds of manual model building using O^29^ and CNS refinement were carried out to incorporate amino acid substitutions, ions, and water molecules, and to rebuild the histone tails. The structure was refined at 2.45 Å to a final crystallographic *R*-factor of 0.229 (*R*_free_ = 0.262) and good geometry.

**Table I:** Data and Refinement Statistics

Data collection	
Space group	P2_1_2_1_2_1_
Cell parameters (Å)	*a* = 106.0, *b* = 182.0, *c* = 109.4
ESRF beamline	ID14-3
Resolution	
Overall (Å)	30–2.45
Outer shell (Å)	2.5–2.45
Completeness (%)^a^	94.6 (84.6)
No. reflections, total	234,968 (10,078)
No. reflections, unique	74,234 (3871)
Redundancy	3.2 (2.6)
R_sym_ (%)^b^	6.2 (48.1)
*I*/σ(*I*)	12.4 (1.7)
Structure Refinement	
*R*_cryst_/*R*_free_ (%)^c^	22.9/26.2
No. of atoms	
Protein	6103
DNA	6021
Water	88
Ions	18
R.m.s.d.bond lengths (Å)/angles (°)	0.009/1.2
Residues in Ramachandran plot (%)	
Most favored/allowed	94.1/5.9
Mean *B*-factors (Å^2^)	
Protein/DNA	51.9/102.2

a Values in parentheses are those for the outer resolution shell.

b *R*_sym_ = Σ_*hkl*_|*I_hkl_* − <*I*>|/Σ*_hkl_I_hkl_*, where *I_hkl_* is the measured intensity of reflections with indices *hkl*.

c *R*_cryst_ = Σ_*hkl*_|*F*_o_ − *F*_c_|/Σ*_hkl_F*_o_, where *F*_o_ and *F*_c_ are the observed and calculated structure factor amplitudes, respectively. *R*_free_ is equal to *R*_cryst_ for a randomly selected 5% subset of reflections not used in the refinement.

## RESULTS AND DISCUSSION

### Structural conservation between the *Xenopus* and *Drosophila* NCPs

As expected, Dm-NCP147 and Xla-NCP147 share a high degree of structural similarity. The histone octamers of the two particles superimpose with an overall root-mean-squares deviation (rmsd) of 0.58 Å for backbone Cα atoms, and 1.00 Å for all atoms including side chains (Table II). These values are approximately half those obtained upon alignment of the yeast and *Xenopus* octamers^23^, consistent with the notion that structural divergence recapitulates phylogeny.^30^–^33^ The individual histone Cα backbones can be aligned with rmsd values of 0.15–0.93 Å. However, these values reduce to 0.15–0.35 Å upon exclusion of a small number of N- or C-terminal residues, where the most significant differences occur. These reduced values correlate well with degree of sequence conservation, the more divergent H2A and H2B histones showing larger rmsd values than the nearly invariant H3 and H4 histones (Table II).

**Table II:** Comparison of Dm-NCP147 and Xla-NCP147 Structures

	RMSD (Å)^a^			Seq. identity (%)^b^
	All structured residues	Excluding termini^c^	Terminal res. excluded^d^	Overall/structured
H2A	0.36 (0.65)	0.33 (0.64)	118–119	88.7/91.6
H2A′	0.67 (1.18)	0.35 (0.84)	12–13	
H2B	0.48 (1.02)	0.28 (0.88)	28–29	82.7/93.6
H2B′	0.49 (0.77)	0.33 (0.68)	29–29	
H3	0.15 (0.64)	0.15 (0.64)	––	99.2/99.0
H3′	0.26 (0.65)	0.17 (0.58)	135	
H4	0.78 (0.95)	0.15 (0.43)	23–24, 102	99.0/100
H4′	0.93 (1.72)	0.18 (0.57)	15–19	
Histone octamer	0.58 (1.00)	0.30 (0.70)	All above	
DNA	0.37 (0.34)			
NCP	0.54 (0.83)	0.32 (0.59)	All above	

a RMSD values for the backbone Cα and DNA phosphate atoms; values in parentheses are for all atoms including side chains.

b Percent sequence identity between *Drosophila* and *Xenopus* histones for all residues, and for residues present in the crystallographic model.

c RMSD values in which structurally most divergent N- and/or C-terminal residues are excluded from the alignment.

d N- and/or C-terminal residues excluded from the alignment.

The conformation of the DNA is essentially identical in the two structures (rmsd = 0.34 Å for all atoms). Unlike the structure of human NCP146, in which the DNA at three superhelix axis locations (SHLs) is shifted relative to Xla-NCP146,^24^ the DNA in Dm-NCP147 remains in register with that of Xla-NCP147.^17^ This is likely a reflection of the higher degree of order generally observed in NCP147 compared to NCP146, irrespective of the source of histones. As in previous NCP structures, a plot of *B*-factor versus base pair shows an oscillating pattern, with minima (40–80 Å^2^) where the DNA contacts histones, and maxima (80–160 Å) at intermediate positions. The manganese and chloride ions identified in Xla-NCP147^18^ are all preserved in our *Drosophila* structure. The entire structure can be superimposed onto that of Xla-NCP147 with an rmsd of 0.82 Å for all protein and DNA atoms, underscoring the high degree of tertiary and quaternary structure conservation.

### Differences in the histone tails

**Figure 1:**
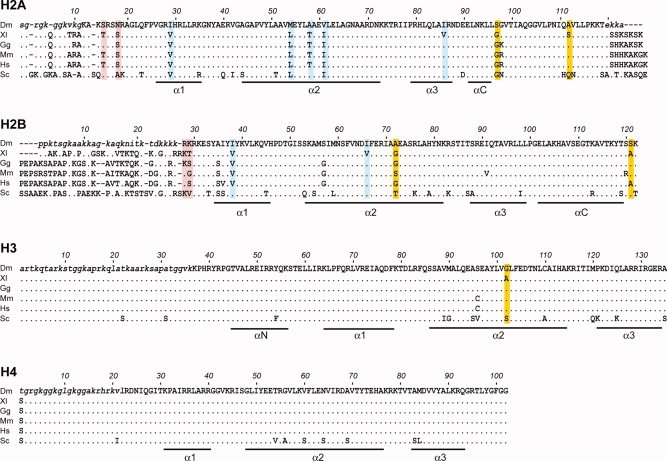
Sequence alignment of histones. Alignment of histones from Drosophila (Dm), Xenopus (Xl), chicken (Gg), mouse (Mm), human (Hs), and yeast (Sc). Drosophila H2A, H2B, H3, H4 sequences correspond to accession codes NP_724343, NP_724342, NP_724345, NP_724344, respectively. Only residues that differ from the Drosophila sequence are shown. Amino acid substitutions that differentiate the Drosophila and Xenopus histone core regions are highlighted in yellow and cyan; those in ordered tail residues are highlighted in pink. Unstructured residues are indicated in lower case.

**Figure 2:**
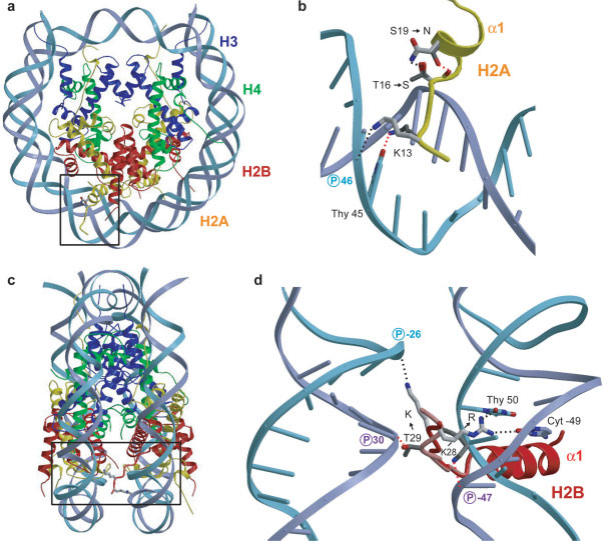
Structural differences within the H2A and H2B histone tails between Dm-NCP147 and Xla-NCP147. (**a**) Dm-NCP viewed along the superhelix showing the location of the N-terminal tail of H2A. (**b**) The N-terminal histone tail of H2A^′^, showing close-up of boxed region in a. Side chains from Drosophila are in light gray; from Xenopus in dark Grey. Hydrogen bonds unique to Drosophila are in black; those unique to Xenopus are in red. Residue substitutions are labelled in the direction from Xenopus to Drosophila. DNA bases are shown as sticks, except for Thy45. The view is slightly rotated relative to that in a. (**c**) Edge view of the NCP showing location of the Nterminal tail of H2B. (**d**) Histone tail of H2B^′^ showing close-up view of boxed region in c. Base atoms is shown for Thy50 and Cyt-49.

A comparison of the Dm- and Xla-NCP147 structures reveals slight differences in the histone tail regions. These probably reflect the inherent structural disorder of the tails, but may also reflect sequence differences (Fig. 1, residues highlighted in pink. Sequence numbering throughout this paper is that of *Xenopus*, which is identical to the *Drosophila* numbering except for H2A). More specifically, in Xla-NCP147, H2A′ residue Lys13 inserts into the minor groove to hydrogen bond with Thy 45 in SHL 4 [Fig. 2(A,B)]. In the *Drosophila* structure, the corresponding Lys residue points more toward the solvent, interacting with the adjacent DNA phosphate group. A few residues away, the *Drosophila* and *Xenopus* H2A sequences diverge at two positions, with *Drosophila* residues Ser and Asn replacing Thr16 and Ser19, respectively. In the *Xenopus* NCP, Ser19 hydrogen bonds to the backbone amide of Thr16, one helical turn away, with the latter in van der Waals contact with the DNA phosphate backbone [Fig. 2(B)]. While the DNA contact is preserved in Dm-NCP147, the intrahelical hydrogen bond is not, its loss compensated by a hydrogen bond gained between the Ser16 and Asn19 side chains [Fig. 2(B)].

In the H2B′ chain of Xla-NCP147 (and -NCP146), residue Thr29 hydrogen bonds with the DNA phosphate backbone (at nucleotide position 30 of chain J) [Fig. 2(C,D)]. In Dm-NCP147, the corresponding Lys29 residue interacts with a phosphate group on the complementary strand (position 26 of chain I). The preceding Arg28 residue adopts a similar orientation as the *Xenopus* Lys28 residue, but inserts more deeply into the minor groove, interacting with the Cyt-49 (chain J) and Thy-50 (chain I). An Arg side chain in a minor groove is a recurrent motif, observed in both the tail and core regions of the various histone chains.^16^ Residues 28 and 29 in the Dm-NCP147 H2B chain are located approximately as in H2B′, but are considerably more disordered. Such variations between otherwise identical chains highlight the structural plasticity of the histone tails.

### Amino acid substitutions in the histone cores

Twelve amino acid substitutions differentiate *Drosophila* from *Xenopus* within the histone core regions. Five substitutions are highly conservative replacements involving solvent-exposed, small (Gly, Ala, or Ser) residues which do not interact with other residues nor with the DNA (Fig. 1, highlighted in yellow). Three of these five substitutions [Gly98 → Ser in H2A; Gly72 → Ala in H2B; and Ala102 → Gly in H3 (written as *Xenopus* → *Drosophila*)] are unlikely to modify interactions between NCP particles, as the residues concerned face solvent regions internal to the octamer core. The other two (Ser113 → Ala in H2A and Ala121 → Ser in H2B) are converse substitutions which localize to the outer face of the NCP; their net effect is the displacement of a single hydroxyl group across the face of the NCP by 45 Å (or 75% of the octamer's diameter), which probably has no more than a modest effect on inter-NCP interactions.

**Figure 3:**
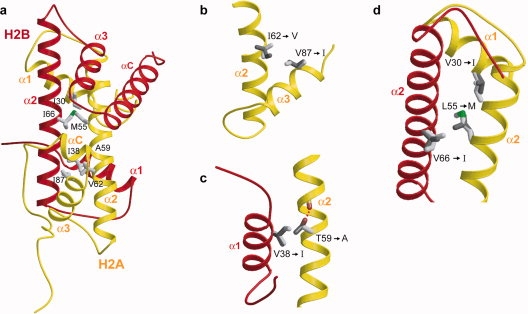
Structural differences in the H2A-H2B dimer between Dm-NCP147 and Xla-NCP147. (**a**) Overview of structured residues in the hydrophobic core of the H2A-H2B dimer that diverge between Xenopus and Drosophila. View is approximately along the pseudodyad. (**b**) Compensatory changes within the hydrophobic core of H2A. Residue substitutions are labeled in the direction from Xenopus to Drosophila. (**c**) Compensatory changes involving two residues in the H2A-H2B dimer interface. The hydrogen bond missing from the Dm structure is in red. (**d**) Compensatory changes involving three residues in the H2A-H2B dimer interface.

The remaining seven substitutions differentiating *Drosophila* from *Xenopus* localize to the hydrophobic core of H2A and to the H2A-H2B dimer interface (Fig. 1, highlighted in cyan). The residues cluster into two groups, located on opposite sides of the pseudodyad [Fig. 3(A)]. Remarkably, all the substitutions are of a mutually compensatory nature. Two substitutions are juxtaposed in histone H2A at positions mediating interactions between the α2 and α3 helices. The converse nature of these substitutions, Ile62 → Val and Val87 → Ile, allows for the net translocation of a methyl group without perturbing the spatial coordinates of the protein backbone [Fig. 3(B)]. A similar phenomenon is observed for *Xenopus* residues H2A-Thr59 and H2B-Val38, which in *Drosophila* are Ala and Ile residues, respectively [Fig. 3(C)]. These two positions are juxtaposed in the dimer interface, such that the gain and loss of a methly group are mutually offset. The Thr59 hydroxyl group forms an intrachain hydrogen bond with the backbone helix α2, and so its loss in *Drosophila* is unlikely to affect dimer stability. Finally, in *Xenopus*, H2A residue Leu55 is sandwiched between Val30 of the same chain and H2B residue Val66. All three positions are substituted in *Drosophila* in such a way as to preserve the volume occupied by side chains in the hydrophobic core: replacement of Leu55 by the more slender Met is countered by replacement of the two valines by bulkier isoleucines [Fig. 3(D)].

Their compensatory nature suggests that these substitutions are unlikely to influence the kinetics or stability of H2A-H2B dimer formation, and hence the dynamics of nucleosome assembly/disassembly. More generally, the phenomenon of counterbalanced substitutions may partly account for the poor correlation observed between sequence conservation and the number of intrachain contacts mediated by histone residues,^34^ because two or more poorly conserved residues may coevolve to preserve inter-residue contacts.

### Patterns in histone evolution

The core histones are among the best-conserved proteins known. The sequence conservation is notably greater in structured (histone-fold) regions than in the histone tails (Fig. 1). Our analysis of *Drosophila* and *Xenopus* NCPs suggests that the sequence divergence in structured residues should have little impact on histone octamer assembly, histone–DNA interactions, or inter-NCP interactions. Clearly, NCP evolution has been tightly constrained since the speciation event that separated the vertebrate and invertebrate clades.

In contrast, yeast and higher eukaryotes exhibit considerably more differences in their histone-fold sequences (Fig. 1), suggesting that histone evolution underwent a burst prior to the appearance of metazoa, only to stagnate thereafter. Although yeast and metazoan NCP structures differ little at the mononucleosomal level, substantial differences in crystal packing interactions suggest that they may exhibit different internucleosomal interactions *in vivo*.^23^ This may be a reflection of the significantly lower requirements for DNA compaction of the much smaller yeast genome compared to that of metazoa.

Mutational studies of histones (both *in vivo* and *invitro*) have made it clear that maintaining nucleosome structure cannot entirely account for the extreme degree of histone sequence conservation. By corollary, sequence changes in histone mutants or variants are of little structural, but of decisive functional consequence. Histones account for a large percentage of the nucleosome's exposed surface–a highly sculpted, differentially charged landscape that interacts with many nuclear factors^35^ and that likely mediates nucleosome–nucleosome interactions to form chromatin higher order structure. Thus, unlike globular proteins, exposed surface residues are exceptionally constrained, and can only mutate if compensatory changes minimize the effects.
